# Tetanus and Strychnia Poisoning—Their Symptoms and Differential Diognosis

**Published:** 1874-06

**Authors:** W. P. Wells

**Affiliations:** Pomeroy, Ohio


### TETANUS AND STRYCHNIA POISONING—THEIR SYMPTOMS AND DIFFERENTIAL DIAGNOSIS.

BY W. P. WELLS, M.D, OF POMEROY, OHIO.

The subject I propose confining my thoughts, and your attention to, for a few moments, is one which not only interests, as a Physician, but has for many years been a source of deep anxiety to the legal as well as the medical profession. I will divide the subject into the following subdivisions and treat each as briefly as possible:

1. Tetanus*
   - *a* Causes
     - Predisposing.
     - Exciting.
   - *b* Symptoms in the order of their occurence.
   - *c* Course.
2. Strychnia Poisoning
   - *a* Doses.
   - *b* Symptoms in their order.
   - *c* Course.
3. Differential Diagnosis
   - *a* Parts involved.
   - *b* Different symptoms.
   - *c* Symptoms in common.
4. Treatment.

1. The predisposing causes of tetanus: *Age*—no period of life is exempt, but infancy and middle life are more subject. *Sex*—males are more liable than females; in the reports of 1,456 cases before me, 992 were males and 464 females, being in the ratio of a little more than two to one. *Habits of life* seem to have no influence; it attacks alike the temperate and intemperate. *Constitutional susceptibility* is not apparent, individuals of any temperament being

*The causes, symptoms and course of tetanus are taken from an abstract of seventy-two cases which occured in Guys Hospital, London.—Guys Hos. Rept., vol. iii., ser. iii.

equally liable. *Climate*—it is more prevalent in warm climate and lowlands near the sea-coast; although season of the year is said to exert no influence. *Race*—the negro seems to be more subject than the white race, even in the same climate.

Of the exciting cause, it may come from the slightest scratch as well as the greatest injury. A scratch on the thumb, a splinter, extracting a tooth, cupping, amputation, abortion, etc. But it occurs most frequently from those injuries which, in point of their *extent* or *persistence*, by continued nervous irritation, are most conducive to shock.

II. Symptoms in the order of their occurrence: 1. Tongue slightly retracted and slight thickness of speech. 2. Rigidity in posterior muscles of the neck, viz.: Sterno-mastoid and trapezius. 3. Muscles of mastication involved; lock-jaw. 4. Muscles of face involved, producing a peculiar tetanic grin, or risus sardonicus. 5. Muscles of pharynx and larynx involved, breathing obstructed slightly, food and drink hard to swallow, and sometimes regurgitated. 6. Diaphragm involved; intense pain from ensiform cartilage to the spine; a feeling as if a cord drawn tightly around the body. 7. Muscles of lower extremities and back involved almost simultaneously. 8. The arms are next involved, the forearm sometimes escapes, and the fingers are rarely involved. 9. The urine retained, or scanty, and high colored. 10. Habitual constipation; if a passage occur it is singularly offensive. 11. Heart's action accelerated, but eventually becomes feeble and irregular. 12. The eyeball fixed and staring, and drawn slightly inwards. 13. Pupil never natural, either dilated or contracted. 14. Temperature above normal standard, sometimes as high as 110½°. 15. Profuse, sour-smelling perspiration.

c. Course of the disease: It generally proceeds in a continuous course of development, affecting the muscular system in the order already named, and generally terminates fatally, in from two to nine days, although we have an instance of death in fifteen* minutes from the time of the injury, and death has been protracted for thirty-two days from the time of the first symptoms. Dupuytren reports a case in which the symptoms subsided for twenty-eight

*Grant's Surgery.

days, and returned after exposure to cold, and proved fatal in a short time. Recovery is preceded by a gradual subsidence of the symptoms, and this happy issue is generally granted to the few that survive the tenth day. It has been stated that the longer the period of incubation, before the commencement of the symptoms, greater is the probability of recovery; and conversely, the shorter the period of incubation, as well as the rapid progress of the symptoms, the more surely fatal is the issue.

2. Strychnia poisoning: *a.* Doses.—This is the hardest portion of my task, as medical aid is generally called, and it is a difficult matter to ascertain how large a dose may be taken, and recovery take place, without medical treatment. However, we have instances of the symptoms of strychnia poisoning from the one-twelfth ($\frac{1}{12}$) of a grain; however, the woman was a delicate female of highly nervous temperament, and it is stated that a dose of seven grains have been recovered from, without medical aid, but the drug is supposed to have been impure. One grain is generally considered a fatal dose, but much depends on the nervous excitability of the individual.

*b.* Symptoms in their order: 1. A feeling of weight and weakness of lower extremities. 2. A sensation, like an electric shock, passing from the toes, up the leg to spinal cord. 3. Slight twitching of the muscles of the leg. 4. Cramping of the legs. After this symptom the following come in such close succession that it is hard to name them in their order, for they are sometimes simultaneous. 5. Rigidity of muscles of the back, arms and legs. 6. Muscles of pharynx and larynx involved. 7. Diaphragm involved. 8. Muscles of mastication involved during exacerbation of the spasm. 9. Urine retained, scanty, or high colored. 10. Constipation, although if recovered from, the bowels easily moved. 11. Temperature above normal; sometimes 110°. 12. Profuse perspiration after the exacerbation of the spasm. 13. Heart's action slightly accelerated during spasm. 14. Pupil of eye dilated or contracted.

*c.* The course is generally very rapid, death sometimes taking place in fifteen minutes, during the exacerbation of the first spasm, but it may be prolonged to eight or ten hours, and the patient may survive several spasms, although very few have been known to survive the fourth spasm.

3. Differential diagnosis: *a.* Parts involved.—Strychnia exerts its influence on the spinal cord, confining its primary action to the lower part of the cord, and extending upward. Tetanus exerts its primary influence on the medulla oblongata, and may extend, by reflex action, through the medium of the sympathetic nerve, to the lower portion of the spinal cord, and then exert its influence upward through the cord. To make this matter clear to your minds, let us examine the anatomy of the parts involved. First, the spinal cord presents two enlargements, the cervical and lumbar; in the cervical enlargement the proportion of gray matter is small, the white largely in excess; in the lumbar enlargement the gray matter is largly in excess, and the white matter smaller in proportion than in the cervical region; the spinal cord ends at the lower portion of the first lumbar vertebra, and terminates in a bulb of gray matter; again, in this bulb terminates the cauda equina, or all the roots of the sacral and lumbar plexus; again, in regard to the gray matter, every collection of gray matter forms a ganglia, or nerve centre. One single cell of gray matter is as much a nerve centre as a thousand. They differ, not in kind, but in degree; its peculiar function is to receive the impression and send out an impulse. Now, as the lower end of the spinal cord is composed of a larger amount of gray matter, it appears quite plausible that it should receive a greater impression and send out a greater impulse.

We are all aware that in paralysis of the bladder and lower extremities that the paralysis yields much quicker and more readily to the influence of strychnia than paralysis of any other portion, and how often we fail when we try to relieve paralysis of the uppe extremities by strychnia. Tetanus, as I said, exerted its influence on the medulla oblongata. Let us trace the symptoms, and examine the muscles involved, and trace the nerves, that supply the muscles, to their deep origin, and we find that they arise in the medulla oblongata, viz.:

Muscles of tongue; nerve hypoglossus; origin, medulla.
Muscles of mastication; nerve, 3d branch of 5th; origin, medulla.
Muscles of face; nerve, facial branch of 7th; origin, medulla.
Muscles of neck, nerve, spinal accessory; origin, one root medulla.
Muscles of eyeball; 3d, 4th and 6th.

At the origin of each of these three nerves fibres can be distinctly traced from the medulla.

*b.* Different symptoms:

| TETANUS. | STRYCHNIA. |
| --- | --- |
| 1. Spasms, tonic or continuous. | 1. Spasms, clonic or intermittent. |
| 2. Symptoms begin in head and neck and proceed downwards. | 2. Symptoms begin in lower extremities and proceed upwards. |
| 3. Muscles of face involved, causing risus sardonicus. | 3. Muscles of face not affected. |
| 4. Eyeball fixed and staring. | 4. Eyeball natural. |
| 5. Arms not involved till the last. | 5. Arms involved early. |
| 6. Continued trismus. | 6. Trismus only during the exacerbation. |

*c.* Symptoms in common: 1. Involvement of the muscles of the pharnyx and larnyx. 2. Diaphragm involved. 3. Temperature above normal. 4. Constipation. 5. Retention of urine, or scanty secretion. 6. Perspiration. 7. Trismus in tetanus continuous in strychnia poisoning during the exacerbation of the spasm only. We will now examine the origin of the nerves that supply the muscles affected above, and we find they are supplied by filaments from both the medulla and spinal cord. 1. Muscles of pharynx and larynx nerve, 8th pair, root from spine and medulla. 2. Diaphragm, nerve, phrenic origin, cervical plexus, receive spinal roots and branches from pneumogastric and hypoglossal. 3. Muscles of mastication—3d branch of fifth origin in medulla. In strychnia the trismus is caused by reflex action through the inter-cranial gaaglia of the sympathetic; each gaaglia receives a sensitive root from the 5th, and the impression is carried through these filaments to the fifth, and it thus sends forth its impulse during the exacerbation of the spasm. 4. The constipation and retention of the urine are due to the influence of the spinal nerves, and does not take the place, to any great extent, in tetanus, until the spine is involved. It is evident that the spinal cord is involved through sympathetic action of the sympathetic nerve; for we find that, in tetanus, the arms are the last part involved. The sympathetic nerve has four inter-cranial gaaglai, viz.: Ophthalmic, Meckels, otic and submaxilary. They all receive sensitive roots from the 5th, the most sensitive nerve in the body; and moter roots from the 3d, 7th, 5th and 9th, in the order above named; and connects intimately with the spinal nerves, in its descent, by two filaments to each spinal root. In both affections the pupil is affected the same. The ciliary muscles of the iris are the highest enclosed with nerves of any muscles in the body, and are under control of the sympathetic, and are contracted or dilated through its influence. The temperature, in tetanus and strychnia, is a problem not easily solved; that it is not on account of inflammation we feel certain. Nothing is easier than to say that the rise in temperature is due to deranged innervation, but in what does the derangement consist, and why should the result be a rise, and not a fall of temperature? That ordinary heat depends on the disintegration of tissue we feel assured; but the hardest of exercise in a limb does not increase the temperature but from 1 to 3°. In all grave injuries to the spinal cord, below the cervical region, we have prostration, decrease of temperature and paralysis generally; while in grave injuries in the cervical region the reverse is the case. One instance, from Morgan's First Principles of Surgery, of fracture of cervical vertebra, the temperature arose to 110°. Dupuytren mentions a case where the skin became very hot. Dr. Packard, of Philadelphia, mentions two cases—one, he says the sensation of heat was almost painful; the other, he says the skin became very hot. It cannot be due to the sympathetic nerve; for its division in the cervical region produces an increase of heat, on the divided side, of from 7* to 10° in this experment. The blood passes through the capillaries with great rapidity, the vessels are dilated, and the arterial blood passes into the veins unchanged. Experiments on the moter cranial nerves produce an exactly opposite effect. When the sympathetic is excited, and not divided, an opposite effect is produced. That this elevation of temperature is produced by the excitability of some one or more of the cranio-spinal nerves I almost feel assured; and, as the 8th pair has the most intimate connection between the spine and medulla, I would suggest that it be experimented on by some one better prepared for the experiment than myself.

4. Treatment: The first great indication in treating both these affections is perfect quiet, and the removal of anything liable to irritate the patient, either in mind or body. It has been shown by the experiments of Bernard, that to take two frogs, and insert strychnia, and immediately decapitate the frogs; irritate one with a feather, and you immediately produce spasm; the other, if left to

*Experiments of Bernard.

itself, shows no sign of spasm. The experiment must be done carefully, for the least jar will produce the spasm. Chloroform has been used successfully in strychnia poisoning by holding the spasms in abeyance until the poison has time to be eliminated; but in tetanus, according to the experiments in Guy's Hospital, it always aggravates the symptoms. The woorari poison, from its action in paralyzing the motor nerves, would be indicated in both these affections. I have noticed one case of tetanus successfully treated by this remedy, by Dr. Spencer Wells, of London; but as yet, on account of the impure state of the drug, the different varieties, and the varying strength of the article, it will not come into general use until we get the drug of a uniform standard. I would also highly recommend the calabar bean. As for other drugs, I have none to recommend, as the whole category have been tried and found wanting.

In the preparation of this paper I have not hesitated to employ the labors of others in the field; but have used a large collection of authorities on the subject, and have endeavored to acknowledge the authority. If, however, I have made any omissions in this respect, it has been done accidently, or because I have considered whatever was thus employed common property of the profession at large.

---

A Good Cement.—A very adhesive cement, and one particularly useful for fastening brass on glass, may be prepared by boiling three parts of resin with one part of caustic soda and five parts of water, thus making a kind of soap, which is mixed with one-half of its weight of plaster of Paris. Zinc white, white lead, or precipitated chalk may be used instead of plaster, but when they are used the cement will be longer in hardening.—*Boston Journal of Chemistry.—Dental Cosmos.*

Acontine First Discovered.—Geigher and Hesse, in 1833, each by his own process, first extracted from the leaves of Aconitum Napellus, a peculiar substance—bitter, amorphous, extremely powerful in its action on the animal economy—to which they gave the name of *Acontine.*

